# Acceptance-Based and ACT-Informed Interventions for Non-Suicidal Self-Injury in Adolescents: A Systematic Review and Exploratory Meta-Analysis

**DOI:** 10.3390/children13070972

**Published:** 2026-07-22

**Authors:** Georgios Giannakopoulos, Afroditi Prassou

**Affiliations:** 1 Department of Child and Adolescent Psychiatry, School of Medicine, National and Kapodistrian University of Athens, “Aghia Sophia” Children’s Hospital, 115 27 Athens, Greece; 2 4th Directorate of Secondary Education of Athens, 171 21 Athens, Greece; aprassou@sch.gr

**Keywords:** non-suicidal self-injury, adolescent, acceptance and commitment therapy, psychological flexibility, emotion regulation, experiential avoidance

## Abstract

**Highlights:**

**What are the main findings?**

- Evidence for primarily ACT, ERITA, and IERITA interventions is promising but remains preliminary.
- The strongest current evidence concerns therapist-guided IERITA evaluated in a single randomized trial, while evidence for primarily ACT interventions and other ERITA formats remains preliminary.

**What are the implications of the main findings?**

- Therapist-guided IERITA may warrant further evaluation as a promising adjunctive or stepped-care option for carefully selected adolescents; however, current evidence is insufficient to support its routine implementation or its use as a replacement for established treatments, particularly DBT-A for high-risk youth.
- Larger preregistered randomized trials with active comparators, longer follow-up, and mediation analyses are needed before any of these interventions can be considered established treatments for adolescent NSSI.

**Abstract:**

Background/Objectives: Non-suicidal self-injury (NSSI) in adolescence is associated with emotion dysregulation, experiential avoidance, psychiatric morbidity, and later suicidal risk. Interventions based primarily on acceptance and commitment therapy (ACT) target psychological flexibility, acceptance, cognitive defusion, and values-based action, whereas emotion regulation individual therapy for adolescents (ERITA) and internet-delivered emotion regulation individual therapy for adolescents (IERITA) principally target emotion dysregulation while incorporating selected acceptance-related and ACT-consistent components, making these approaches theoretically relevant to adolescent NSSI. Methods: We conducted a systematic review and exploratory meta-analysis informed by the Preferred Reporting Items for Systematic Reviews and Meta-Analyses (PRISMA) 2020 statement. PubMed, Scopus, Web of Science, and EBSCO were searched on 10 April 2026. Eligible studies included adolescents or predominantly adolescent samples in which NSSI was the central clinical target or NSSI-specific outcomes were separately extractable and evaluated primarily ACT, ACT-informed, acceptance-based, ERITA, or IERITA interventions. Quantitative syntheses were restricted to controlled studies with extractable data. Embase, the Cochrane Central Register of Controlled Trials (CENTRAL), trial registries, and dedicated regional or gray literature sources were not searched; 15 reports remained unretrieved, so the evidence base may be incomplete. Results: Six full-text primary adolescent NSSI intervention studies met inclusion criteria: two controlled primarily ACT studies, one randomized ERITA feasibility trial, two uncontrolled ERITA feasibility/open studies, and one randomized IERITA trial. The strongest single-study evidence came from therapist-guided IERITA, which reduced masked assessor-rated NSSI frequency more than treatment as usual, with an incidence rate ratio of 0.34 (95% confidence interval [CI] 0.20 to 0.57). Two-study exploratory random-effects syntheses yielded estimates in the direction of benefit for continuous NSSI outcomes, Hedges’ g = −0.45 (95% CI −0.85 to −0.05; k = 2), and process outcomes, Hedges’ g = 1.25 (95% CI 0.83 to 1.68; k = 2). These estimates are hypothesis-generating and imprecise as estimates of a generalizable treatment effect because each synthesis contained only two clinically and methodologically heterogeneous studies. Although I^2^ was 0% in both syntheses, these estimates are highly uncertain with only two studies and should not be interpreted as evidence of true homogeneity. Using the Grading of Recommendations Assessment, Development and Evaluation (GRADE) approach, certainty was moderate for the single masked clinician-rated IERITA outcome and very low for the pooled continuous NSSI and process-outcome evidence. Conclusions: Primarily ACT, ERITA, and IERITA interventions may be promising for adolescent NSSI, but the evidence remains preliminary and mostly of very low certainty. The pooled estimates are exploratory, hypothesis-generating, and imprecise and should not be interpreted as evidence of treatment efficacy. The strongest single-study evidence concerns therapist-guided IERITA and was rated as moderate in certainty, whereas the evidence for primarily ACT interventions and pooled process outcomes was very low in certainty and requires confirmation in larger preregistered randomized trials with standardized outcomes, longer follow-up, active comparators, and process measures.

## 1. Introduction

Non-suicidal self-injury (NSSI) is commonly defined as the deliberate, direct destruction or alteration of one’s own body tissue without suicidal intent and for purposes not socially sanctioned [1]. Although definitions vary across countries and the literature, NSSI is generally distinguished from suicide attempts by the absence of intent to die, while remaining clinically important because it predicts subsequent suicidal ideation and behavior [2,3]. The developmental timing of NSSI is especially important: onset often occurs in early or middle adolescence, and the behavior may become recurrent during a period characterized by heightened affective reactivity, increased peer salience, identity development, and still-maturing regulatory capacities [4,5,6].

A major functional account of NSSI is that it serves as an emotion regulation strategy. Adolescents who self-injure frequently report that the behavior reduces overwhelming affect, interrupts escalating distress, converts diffuse psychological pain into concrete bodily sensation, or provides short-term relief from shame, anger, emptiness, anxiety, or self-criticism [7,8,9,10]. In behavioral terms, this relief may negatively reinforce self-injury, thereby increasing the likelihood of repetition [11,12]. The experiential avoidance model of deliberate self-harm proposes that self-injury may be maintained by escape from aversive private experiences, such as thoughts, emotions, memories, bodily sensations, and urges [13,14]. A systematic review of self-harm, emotion regulation, and experiential avoidance similarly concluded that the literature tentatively supports a role for experiential avoidance, while repeatedly identifying poor emotion regulation as a core reason for self-harm engagement [13].

Acceptance and commitment therapy (ACT) is therefore theoretically well aligned with adolescent NSSI [7,15]. ACT conceptualizes psychopathology through the lens of psychological inflexibility, including experiential avoidance, cognitive fusion, dominance of the conceptualized past or future, attachment to a narrow self-concept, values ambiguity, and ineffective action [16]. Rather than attempting to eliminate painful internal experiences, ACT aims to increase psychological flexibility: the ability to contact the present moment and act in accordance with values even in the presence of distress [17]. In adolescent NSSI, this model suggests that self-injury urges may be approached as internal events to be noticed, accepted, and defused from, rather than acted upon automatically [18,19]. Values-based committed action may then provide alternative behavioral pathways when distress is high [19,20].

Despite this conceptual fit, the evidence base for ACT in adolescent NSSI remains less developed than that for dialectical behavior therapy for adolescents (DBT-A). Reviews of self-harm interventions in youth continue to identify DBT-A as the best-supported approach for repeated self-harm and suicidal ideation, while other interventions remain preliminary [21,22,23,24]. At the same time, emerging interventions such as emotion regulation individual therapy for adolescents (ERITA) and internet-delivered emotion regulation individual therapy for adolescents (IERITA) explicitly target emotion dysregulation and include acceptance-related and ACT-consistent components, including emotional awareness, acceptance, validation, parent involvement, and behavior in the presence of painful affect [25,26,27].

The present review was designed to synthesize this emerging evidence base. We focus not only on whether interventions reduce NSSI but also on whether they improve processes that theoretically maintain NSSI: emotion dysregulation, experiential avoidance, cognitive fusion, emotional inhibition, and psychological inflexibility. We therefore reviewed primarily ACT, ACT-informed, acceptance-based, ERITA, and IERITA interventions for adolescent NSSI, with quantitative synthesis where data permitted.

## 2. Materials and Methods

### 2.1. Search Strategy and Selection Criteria

This systematic review and exploratory meta-analysis was conducted according to the Preferred Reporting Items for Systematic Reviews and Meta-Analyses (PRISMA) 2020 statement [28]. The review registration is available in the Open Science Framework (OSF; https://doi.org/10.17605/OSF.IO/2AK69). Because registration occurred after completion of the electronic searches, the OSF record should be considered a retrospective registration record rather than a prospective preregistration. The broad clinical question, the focus on adolescents with NSSI, the inclusion of ACT and acceptance-related interventions, and the designation of NSSI frequency or severity as the primary outcome were established before formal screening. Emotion regulation and ACT-related process outcomes were also identified in advance as secondary outcomes, although the specific measures available for synthesis could not be determined before study retrieval. The OSF record was created after the electronic searches and after the available literature had begun to be examined; it therefore documents the review retrospectively and did not prospectively constrain all methodological decisions. Operational eligibility rules, the detailed extraction framework, the risk-of-bias approach, and the quantitative synthesis plan were finalized after the designs and reporting formats of the potentially eligible studies were known.

Post hoc methodological adaptations included treating mixed adolescent/young-adult or mixed-behavior studies as contextual evidence unless separate adolescent NSSI data were available; distinguishing primarily ACT, ACT-informed, acceptance-based, ERITA, and IERITA intervention categories; restricting quantitative synthesis to controlled studies with extractable numerical data; selecting one continuous NSSI outcome per study for the primary standardized mean difference synthesis; reporting the model-based incidence rate ratio from the largest randomized trial separately; summarizing mediation findings narratively when they could not be expressed as comparable standardized mean differences; expanding the risk-of-bias table to include study-specific domain-level justifications and adding an outcome-level certainty-of-evidence assessment; and conducting a sensitivity synthesis that included a second, non-independent outcome from Yuan et al. The risk-of-bias frameworks described in Section 2.3 and the DerSimonian–Laird random-effects model were also specified after the eligible study designs and available outcome structures were known. These decisions were not prospectively preregistered and are therefore reported as post hoc methodological adaptations. No change was made to the broad clinical question or to the designation of NSSI as the primary outcome. The completed PRISMA 2020 checklist is provided as Supplementary Table S1.

Searches were conducted in PubMed, Scopus, Web of Science, and EBSCO on 10 April 2026. The search strategy was intentionally broad and combined three domains: ACT and related mechanisms/interventions; NSSI, self-harm, and self-injury terms; and adolescent/youth terms. No language restriction was applied at the search stage. The search was limited to four major biomedical and multidisciplinary bibliographic sources and did not comprehensively cover gray, registered, dissertation, or regional literature. Embase, the Cochrane Central Register of Controlled Trials (CENTRAL), trial registries, and regional bibliographic databases were not searched directly, and no dedicated dissertation or gray literature search was undertaken. Given the small and geographically dispersed evidence base, the review should not be interpreted as exhaustive.

The PubMed search was ((“Acceptance and Commitment Therapy”[Mesh] OR “acceptance and commitment therap*”[tiab] OR “acceptance commitment therap*”[tiab] OR “ACT-based”[tiab] OR “ACT informed”[tiab] OR “ACT-informed”[tiab] OR “acceptance-based”[tiab] OR “psychological flexibil*”[tiab] OR “experiential avoidance”[tiab] OR “cognitive fusion”[tiab] OR ERITA[tiab] OR IERITA[tiab] OR “Emotion Regulation Individual Therapy for Adolescents”[tiab]) AND (“Non-Suicidal Self-Injury”[tiab] OR “Nonsuicidal Self-Injury”[tiab] OR NSSI[tiab] OR “self-injur*”[tiab] OR “self injur*”[tiab] OR “self-harm”[tiab] OR “self harm”[tiab] OR “deliberate self-harm”[tiab] OR “deliberate self harm”[tiab] OR “self-mutilat*”[tiab] OR “Self-Injurious Behavior”[Mesh] OR “Self Mutilation”[Mesh]) AND (adolescen*[tiab] OR teen*[tiab] OR youth[tiab] OR youths[tiab] OR “young people”[tiab] OR “young person*”[tiab] OR child*[tiab] OR pediatric*[tiab] OR paediatric*[tiab] OR “Adolescent”[Mesh] OR “Child”[Mesh])) NOT (animals[mh] NOT humans[mh])

The Scopus search was TITLE-ABS-KEY (“acceptance and commitment therap*” OR “acceptance commitment therap*” OR “ACT-based” OR “ACT informed” OR “ACT-informed” OR “acceptance-based” OR “acceptance based” OR “psychological flexib*” OR “experiential avoidance” OR “cognitive fusion” OR ERITA OR IERITA OR “emotion regulation individual therapy” OR “internet-delivered emotion regulation individual therapy” OR “internet based emotion regulation individual therapy” OR “destructive experiential avoidance”) AND TITLE-ABS-KEY (“non-suicidal self-injur*” OR “nonsuicidal self-injur*” OR NSSI OR “self-injur*” OR “self injur*” OR “self-harm” OR “self harm” OR “deliberate self-harm” OR “deliberate self harm” OR “self-mutilat*” OR “self mutilat*” OR “self-destructive behavio*” OR “self destructive behavio*”) AND TITLE-ABS-KEY (adolescen* OR teen* OR youth OR youths OR “young people” OR “young person*” OR child* OR pediatric* OR paediatric*)

The Web of Science Core Collection search was TS = (“acceptance and commitment therap*” OR “acceptance commitment therap*” OR “ACT-based” OR “ACT informed” OR “ACT-informed” OR “acceptance-based” OR “acceptance based” OR “psychological flexib*” OR “experiential avoidance” OR “cognitive fusion” OR ERITA OR IERITA OR “emotion regulation individual therapy” OR “internet-delivered emotion regulation individual therapy” OR “internet based emotion regulation individual therapy” OR “destructive experiential avoidance”) AND TS = (“non-suicidal self-injur*” OR “nonsuicidal self-injur*” OR NSSI OR “self-injur*” OR “self injur*” OR “self-harm” OR “self harm” OR “deliberate self-harm” OR “deliberate self harm” OR “self-mutilat*” OR “self mutilat*” OR “self-destructive behavio*” OR “self destructive behavio*”) AND TS = (adolescen* OR teen* OR youth OR youths OR “young people” OR “young person*” OR child* OR pediatric* OR paediatric*)

The EBSCO search was (“acceptance and commitment therap*” OR “acceptance commitment therap*” OR “ACT-based” OR “ACT informed” OR “ACT-informed”) AND (“non-suicidal self-injur*” OR “nonsuicidal self-injur*” OR NSSI OR “self-injur*” OR “self-harm” OR “self injury” OR “self harm”) AND (adolescen* OR teen* OR youth OR child*)

The variants “ACT-based”, “ACT informed”, “ACT-informed”, and “acceptance-based” were retained in the search strings to maximize retrieval sensitivity and should not be interpreted as equivalent analytic intervention categories. The EBSCO search yielded 92 records, which were exported in two batches because of platform export limits and were then combined before deduplication.

Studies were eligible if they included adolescents or predominantly adolescent samples in which NSSI or NSSI disorder was the central clinical target, or if a broader self-harm sample reported NSSI-specific outcomes separately; evaluated an intervention subsequently classified as primarily ACT, ACT-informed, acceptance-based, ERITA, or IERITA; and reported relevant intervention outcomes. Studies reporting only a composite self-harm outcome that combined non-suicidal and suicidal self-harm without separately extractable NSSI data were not eligible for the primary systematic review or quantitative synthesis. Randomized controlled trials, feasibility randomized trials, controlled non-randomized studies, quasi-experimental studies, and uncontrolled feasibility/open studies were eligible for the systematic review. Quantitative synthesis was restricted to controlled studies with extractable numerical outcome data. Reviews, protocols, secondary analyses, qualitative studies, adult-only studies, DBT-only studies that did not meet any of the eligible intervention classifications, and purely observational/mechanistic studies were excluded from the primary efficacy synthesis.

Studies with mixed adolescent/young-adult samples, mixed self-harm outcomes, or mixed destructive experiential avoidance behaviors were not counted as primary adolescent NSSI intervention studies unless adolescent NSSI was the central clinical target and NSSI-specific data were reported separately for the eligible population. Composite outcomes combining NSSI with suicidal self-harm were not entered into the quantitative syntheses. Studies that did not permit this distinction were summarized only as contextual evidence when clinically relevant.

### 2.2. Screening Procedure and Data Extraction

Two reviewers independently screened all titles and abstracts against the stated eligibility criteria. Full texts were sought for all records retained after the initial screening, and the reports successfully retrieved were independently assessed by the same two reviewers. At both stages, each reviewer made an eligibility judgment before discussion. Disagreements concerning the population, intervention classification, relevance to NSSI, study design, or outcome extractability were resolved through discussion and consensus before the final inclusion decision was made. When the full text could not be retrieved despite institutional-access and open-web searches, the report was classified as not retrieved and was not assessed for eligibility or included in any quantitative synthesis. Fifteen reports remained in this category. No report was included in the review, and no effect size was extracted, solely on the basis of abstract-level information. Direct searches of regional bibliographic or full-text databases, including CNKI and Wanfang for Chinese literature and SID and Magiran for Persian literature, were not undertaken.

For each included primary study, two reviewers independently extracted the following variables using a standardized extraction form: country, study design, sample size, age range, sex distribution when reported, inclusion criteria, intervention type, comparator, treatment duration, outcome measures, timing of assessment, NSSI outcome, emotion-regulation or psychological-flexibility outcomes, follow-up period, adverse events, worsening NSSI or other self-harm, suicidal ideation or attempts, crisis or emergency service use, hospitalization, treatment escalation, treatment discontinuation or dropout and the reported reasons, and main results. When a safety outcome was not reported, it was recorded as not reported rather than interpreted as evidence that no event occurred.

For each outcome considered for quantitative synthesis, the reviewers additionally extracted the arm-specific means, standard deviations, analysis sample sizes, assessment time point, comparator, direction of scoring, and whether the reported values represented post-treatment scores or change from baseline. When the primary study reported a model-based effect rather than raw continuous data, the reported metric, model specification where available, and analysis sample were recorded separately. These numerical inputs are presented in Supplementary Table S2.

For synthesis, terminology was standardized according to the degree of documented ACT specificity. Interventions were classified into five conceptually distinct categories: (1) primarily ACT interventions, when the treatment was explicitly presented as ACT and organized predominantly around core ACT processes; (2) ACT-informed interventions, when the treatment was not presented as a complete ACT protocol but was explicitly grounded in the ACT model and incorporated multiple core ACT processes; (3) acceptance-based interventions, when acceptance was a central treatment component but the publication did not provide sufficient evidence of broader ACT theoretical or procedural fidelity; (4) ERITA interventions, when the study authors identified the treatment as Emotion Regulation Individual Therapy for Adolescents, whether delivered face-to-face or online; and (5) IERITA interventions, when the treatment was explicitly identified as the internet-delivered IERITA protocol. The phrase “ACT-consistent element” was used only to describe an individual component or mechanism compatible with ACT and did not denote an ACT or ACT-informed intervention. “ACT-based” was not used as an analytic intervention category because it does not specify the extent of ACT content or fidelity; it was retained only within the verbatim search strategies as a retrieval term. ERITA and IERITA were treated as related but distinct multicomponent emotion-regulation interventions incorporating selected acceptance-related and ACT-consistent elements alongside additional behavioral and emotion regulation components. These categories were included within the same review because they addressed the same clinical target and overlapping hypothesized mechanisms, but they were not assumed to be therapeutically equivalent. Findings were interpreted by intervention category, and any cross-category quantitative synthesis was considered exploratory and was not interpreted as evidence of ACT-specific efficacy. Classification was based on the intervention identity, theoretical rationale, and treatment components reported by the study authors; ACT fidelity was not independently assessed. Study investigators were not contacted to obtain or clarify missing numerical outcome data.

### 2.3. Risk of Bias and Certainty of Evidence Assessment

Risk of bias was assessed independently by two reviewers using the revised Cochrane risk-of-bias tool for randomized trials (RoB 2) and the Risk of Bias in Non-randomized Studies of Interventions (ROBINS-I) framework for non-randomized or uncontrolled studies [29,30]. Disagreements were resolved through discussion and consensus. Because the review included heterogeneous study designs, the studies were not collapsed into a single numerical risk-of-bias score. Instead, judgments were made across the following domains: randomization or confounding, selection or allocation, missing data, outcome measurement, selective reporting, and overall causal interpretability. Domain-level judgments and brief study-specific justifications were recorded for each study. Uncontrolled open studies were considered at critical risk of bias for causal efficacy because the absence of a comparator precludes separation of intervention effects from regression to the mean, spontaneous improvement, concurrent treatment, or expectancy effects.

Certainty of evidence was assessed at the outcome level using the Grading of Recommendations Assessment, Development and Evaluation (GRADE) approach [31]. Two reviewers independently considered risk of bias, inconsistency, indirectness, imprecision, and publication bias and assigned one of four certainty ratings: high, moderate, low, or very low. Disagreements were resolved through discussion and consensus. Certainty was assessed for each outcome-level body of evidence rather than inferred solely from the design of individual studies. No upgrading for large effects was applied because the largest estimates arose from small or methodologically limited studies. The GRADE assessment was added during revision and was not prospectively specified in the OSF record. Detailed outcome-level judgments are presented in Supplementary Table S3.

### 2.4. Meta-Analyses

The primary outcome was NSSI frequency or severity. Only outcomes explicitly measuring NSSI were eligible for quantitative synthesis. Composite self-harm outcomes that combined NSSI with suicide attempts or self-injury involving suicidal intent were excluded unless NSSI-specific results could be separated. Secondary outcomes included emotion regulation, cognitive fusion, psychological flexibility-related processes, emotional inhibition, depressive/anxiety/stress symptoms, global functioning, feasibility, and acceptability. For continuous outcomes, Hedges’ g was calculated from either post-treatment group means and standard deviations or directly reported group-specific change-score means and standard deviations. Let $n_{I}$, $M_{I}$, and ${SD}_{I}$ denote the intervention-group sample size, mean, and standard deviation, respectively, and $n_{C}$, $M_{C}$, and $SD_{C}$ the corresponding comparator-group values. The pooled standard deviation was calculated as

$$SD_{p}=\sqrt{\left(\frac{\left(n_{I}-1\right)SD_{I}^{2}+\left(n_{C}-1\right)SD_{C}^{2}}{n_{I}+n_{C}-2}\right)}$$

Cohen’s d was calculated as

$$d=\frac{M_{I}-M_{C}}{SD_{p}}$$

Cohen’s d was corrected for small-sample bias using Hedges’ correction factor:

$$J=1-\frac{3}{4df-1}$$

where

$$df=n_{I}+n_{C}-2$$

such that

g=Jd

The sampling variance was calculated as

$$V_{g}=\frac{n_{I}+n_{C}}{n_{I}\cdot n_{C}}+\frac{g^{2}}{2\left(n_{I}+n_{C}-2\right)}$$

The 95% confidence interval was calculated as

$$g\pm 1.96\sqrt{V_{g}}$$

For the Yuan et al. [15] outcomes, the published group-specific T0–T2 change-score means and standard deviations were used directly; therefore, no baseline–post-treatment correlation was estimated or imputed. For Morthorst et al. [27] and Falahati et al. [32], post-treatment means and standard deviations were used. NSSI effects were calculated as intervention minus comparator, so negative values indicated a lower NSSI frequency or severity in the intervention group. Process outcomes were oriented so that positive values indicated improvement. No standardized mean difference was derived from *p* values, t statistics, medians, interquartile ranges, or incidence rate ratios. The model-based incidence rate ratio reported by Bjureberg et al. [33] was extracted unchanged and was not transformed into Hedges’ g or included in the SMD meta-analysis. All numerical inputs and calculation decisions are reported in Supplementary Table S2.

Exploratory pooling was undertaken because the contributing controlled studies addressed the same broad clinical question, evaluated interventions sharing acceptance-related or ACT-consistent therapeutic elements, and reported conceptually aligned NSSI or emotion regulation outcomes that could be expressed on a common standardized mean difference scale. The purpose was to summarize the direction and approximate magnitude of the available signals, not to assume that the interventions, populations, comparators, measures, or underlying treatment effects were equivalent. NSSI outcomes and process outcomes were therefore synthesized separately, only one continuous NSSI outcome per study was included in the primary synthesis, and the model-based count outcome from the largest randomized trial was reported separately because it was not commensurable with the standardized mean differences. The primary continuous NSSI synthesis therefore combined one randomized ERITA feasibility trial with one retrospective non-randomized primarily ACT study, whereas the process–outcome synthesis combined two methodologically limited primarily ACT studies assessing different emotion regulation constructs. Standardization placed the outcomes on a common numerical scale but did not make the studies clinically or methodologically exchangeable. Pooling was retained only as a descriptive summary of the direction and approximate magnitude of the available signals, and individual-study and intervention-specific findings were given priority in interpretation.

Random-effects models were used to allow the underlying effects to vary across studies because the included studies differed in intervention content, delivery format, study design, outcome measurement, comparator, and assessment timing. The use of a random-effects model did not remove or resolve this clinical and methodological heterogeneity. Study-level effect sizes were calculated from the reported summary statistics using the formulas described above. Random-effects meta-analyses were conducted using IBM SPSS Statistics, version 31.0 (IBM Corp., Armonk, NY, USA), with between-study variance estimated using the DerSimonian–Laird method. Because each synthesis contained only two or three effects, the pooled estimates were treated as exploratory, descriptive, and hypothesis-generating rather than as confirmatory evidence of efficacy. All pooled and sensitivity analyses were post hoc and should be interpreted accordingly given that the quantitative synthesis plan was finalized after the available study designs and outcome structures were known. The I^2^ statistic was reported descriptively; however, with such a small number of studies, both I^2^ and the estimated between-study variance have very low precision. In particular, an observed I^2^ of 0% should not be interpreted as demonstrating that the contributing studies are clinically or methodologically homogeneous. Funnel plots and formal tests of publication bias were not considered reliable. The large Swedish randomized controlled trial (RCT) by Bjureberg et al. [33] reported a model-based incidence rate ratio for NSSI frequency; this result was reported separately and not forced into a standardized mean difference (SMD) framework.

### 2.5. Study Selection Flow

Database searches identified 333 records: PubMed (*n* = 25), Scopus (*n* = 98), Web of Science (*n* = 118), and EBSCO (*n* = 92). After removal of 103 duplicate records, 230 records were screened. One hundred and eighty records were excluded at title/abstract screening. Fifty reports were sought for retrieval; 15 potentially relevant regional, non-mainstream, or non-English reports could not be retrieved in full text and therefore remained unassessed for eligibility. Thirty-five full-text reports were assessed for eligibility, and 29 were excluded because they evaluated an intervention outside the prespecified eligible categories, involved the wrong population, were reviews/protocols/secondary analyses/qualitative reports, were observational/mechanistic studies without an intervention, or involved a mixed age/behavior sample not eligible for the main adolescent NSSI synthesis.

**Figure 1:**
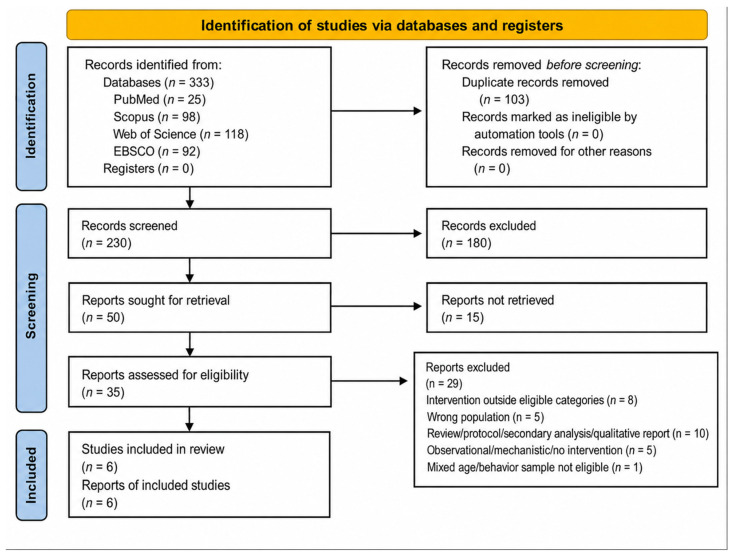
Preferred Reporting Items for Systematic Reviews and Meta-Analyses (PRISMA) 2020 flow diagram.

Six full-text primary adolescent NSSI intervention studies were included in the systematic review. Four controlled studies contributed at least one quantitative estimate: Bjureberg et al. [33] contributed a separate model-based count outcome; Morthorst et al. [27] and Yuan et al. [15] contributed to the primary continuous NSSI SMD synthesis; and Yuan et al. [15] and Falahati et al. [32] contributed to the process-outcome synthesis. One additional ACT feasibility study, the Acceptance and Commitment Therapy for Destructive Experiential Avoidance (ACT-DEA) study by Na et al. [34], targeted destructive experiential avoidance in a mixed adolescent/young-adult sample and was summarized as contextual evidence but was not included in the main synthesis. Details of the study selection process are presented in Figure 1.

## 3. Results

### 3.1. Systematic Review Results

Six primary adolescent NSSI intervention studies met the inclusion criteria. To avoid conflating treatments with different levels of ACT specificity, the evidence was described separately as primarily ACT, ACT-informed, acceptance-based, ERITA, and IERITA. Two controlled studies were classified as primarily ACT: Yuan et al. [15] and Falahati et al. [32]. No included primary study met the criteria for a standalone ACT-informed or acceptance-based intervention. ERITA evidence comprised the open online pilot by Bjureberg et al. [25], the randomized feasibility trial by Morthorst et al. [27], and the face-to-face open feasibility study by Bjureberg et al. [35]. IERITA evidence comprised the three-site randomized trial by Bjureberg et al. [33]. ERITA and IERITA were treated as related but distinct multicomponent emotion-regulation interventions, not as complete ACT protocols or as evidence of ACT-specific efficacy. Accordingly, the stronger IERITA finding was not generalized to primarily ACT, ACT-informed, acceptance-based, or ERITA interventions. Table 1 provides a comparative summary of the intervention characteristics, study design, overall methodological appraisal, and principal findings of the six primary studies.

Bjureberg et al. [33] conducted the strongest efficacy study: a three-site, single-masked randomized clinical trial of IERITA plus treatment as usual (TAU) versus TAU alone in 166 adolescents aged 13–17 years with NSSI disorder. IERITA consisted of a 12-week therapist-guided internet-delivered treatment with adolescent and parent modules. The primary outcome was NSSI frequency measured with the youth version of the Deliberate Self-Harm Inventory, assessed by both self-report and masked clinicians. The intervention significantly reduced masked assessor-rated NSSI frequency relative to TAU, with an incidence rate ratio of 0.34 (95% confidence interval [CI] 0.20 to 0.57) and maintenance at three-month follow-up.

Morthorst et al. [27] conducted a Danish randomized feasibility trial. Thirty adolescents aged 13–17 years with recurrent NSSI were randomized to internet-based ERITA plus TAU or TAU alone. The trial primarily evaluated feasibility rather than efficacy. Follow-up completion and intervention compliance were high, but the exploratory clinical outcome, NSSI episodes measured with the Deliberate Self-Harm Inventory–Youth version (DSHI-Y), did not differ significantly between groups. The direction of the effect favored ERITA, but the sample was small, and the confidence interval was wide.

Yuan et al. [15] evaluated ACT plus routine psychological support versus routine psychological support alone in 72 adolescents aged 13–18 years meeting NSSI criteria according to DSM-5 [36]. The study was retrospective and controlled rather than randomized. The ACT program consisted of nine 60 min group sessions over six weeks. ACT was associated with greater reductions in adolescent NSSI behavior and function questionnaire scores, greater improvement in positive emotion regulation, and greater reduction in negative emotion regulation relative to routine support.

Falahati et al. [32] evaluated ACT in a semi-experimental controlled study of 30 preadolescent/early adolescent girls aged 11–13 years with NSSI. The intervention consisted of eight weekly 90 min ACT sessions. The outcomes were cognitive emotion regulation and emotional inhibition, rather than direct NSSI frequency. Both process outcomes improved more in the ACT group than in the control condition.

Bjureberg et al. [35] reported a face-to-face ERITA feasibility study in 17 girls aged 13–17 years with NSSI disorder. The uncontrolled open design precludes causal inference, but treatment credibility, alliance, attendance, and completion were acceptable, and pre-post improvements were observed in NSSI frequency, NSSI versatility, emotion regulation difficulties, self-destructive behaviors, and global functioning. Bjureberg et al. [25] extended this work to an online ERITA open pilot in 25 adolescents, reporting a 55% reduction in past-month NSSI frequency from pre-treatment to post-treatment, further improvement at three-month follow-up, and maintenance at six-month follow-up.

Na et al. [34] evaluated ACT-DEA, a six-session ACT program targeting destructive experiential avoidance behaviors in 20 participants aged 15–25 years. Deliberate self-harm was common, but the sample included both adolescents and young adults and targeted mixed destructive experiential avoidance behaviors, including self-harm and addiction-related behaviors. For this reason, the study was summarized as contextual ACT feasibility evidence but was not counted as a primary adolescent NSSI intervention study and was not included in the main synthesis.

Across the six primary studies, the findings were generally in the direction of reduced NSSI or improvement in theoretically relevant emotion regulation processes, but the strength and interpretability of the evidence differed substantially. The clearest controlled NSSI signal came from the single larger IERITA randomized trial. The ERITA studies mainly provided feasibility, acceptability, and preliminary pre–post evidence, whereas the two primarily ACT studies reported favorable NSSI-related or process outcomes under retrospective or semi-experimental conditions. The studies differed in intervention content, delivery format, parental involvement, comparator condition, outcome measurement, and follow-up duration. Overall methodological quality ranged from a comparatively stronger randomized trial with masked outcome assessment to small feasibility, retrospective, semi-experimental, and uncontrolled studies with serious or critical limitations for causal inference. Taken together, the studies support further investigation but do not provide a sufficiently robust or homogeneous basis for a definitive cross-intervention efficacy conclusion.

### 3.2. Risk of Bias and Certainty of Evidence Results

The risk-of-bias assessment is summarized in Table 2. The Bjureberg et al. [33] RCT had the strongest design, with random allocation, masked clinician-rated outcome assessment, a manualized intervention, and clearly defined NSSI disorder eligibility. Remaining risk-of-bias concerns related mainly to incomplete outcome data and the inability to blind participants and treatment providers, although masked assessment of the primary NSSI outcome reduced concern about outcome measurement bias. Short follow-up, the predominantly female sample, and the intervention-specific nature of IERITA were considered separately in the GRADE assessment. The Morthorst et al. [27] feasibility RCT had randomization and trial procedures appropriate for feasibility evaluation, but it was not powered for clinical efficacy. Yuan et al. [15] provided important evidence for primarily ACT interventions but had a serious risk of confounding because allocation was based on the management method in a retrospective controlled design. Falahati et al. [32] was limited by small sample size, unclear allocation details, absence of active comparator, and lack of direct NSSI-frequency outcome. The two open ERITA studies were considered to have a critical risk of bias for causal efficacy because they lacked control groups.

The expanded domain-level assessment showed that the two uncontrolled ERITA studies were at a critical risk of bias for causal efficacy, whereas the two non-randomized ACT studies had serious limitations arising principally from confounding, allocation, and unblinded outcome measurement. The randomized ERITA feasibility trial and the IERITA efficacy trial had stronger designs, although the feasibility trial was small and the larger IERITA trial retained some concerns related to incomplete outcome data, unblinded treatment delivery, and short follow-up.

The GRADE assessment rated the certainty of evidence as moderate for the masked clinician-rated NSSI-frequency outcome from the single IERITA randomized trial. Certainty was very low for the pooled continuous NSSI outcome, the pooled process outcome, and the feasibility and acceptability evidence. The principal reasons for downgrading were serious risk of bias in the non-randomized studies, indirectness arising from differences in interventions, comparators, populations, and outcome measures, imprecision arising from very small evidence bodies, and suspected publication and availability bias. Safety evidence was insufficient for a certainty rating. Detailed outcome-level judgments are presented in Supplementary Table S3.

### 3.3. Effects on NSSI Frequency or Severity

**Figure 2:**
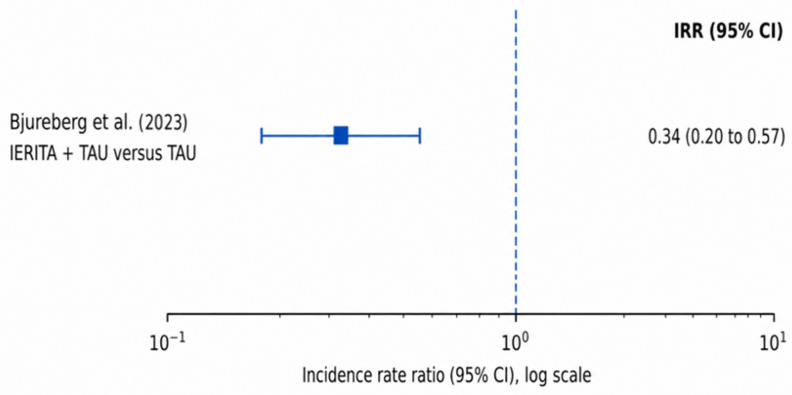
Model-based non-suicidal self-injury count outcome from the largest randomized controlled trial by Bjureberg et al. [33]. Note. The blue square represents the IRR point estimate, the horizontal blue line represents the 95% CI, and the vertical dashed blue line marks the null value (IRR = 1.00). CI = confidence interval; IERITA = internet-delivered emotion regulation individual therapy for adolescents; IRR = incidence rate ratio; TAU = treatment as usual. Values below 1.00 favor IERITA + TAU.

The clearest controlled efficacy signal came from Bjureberg et al. [33]. IERITA plus TAU produced an 82% reduction in masked assessor-rated NSSI frequency compared with a 47% reduction in TAU alone. The incidence rate ratio was 0.34 (95% CI 0.20 to 0.57), indicating significantly fewer NSSI episodes in the intervention arm. Because this was a model-based count outcome, it was reported separately rather than pooled with standardized mean differences (Figure 2).

The randomized feasibility trial by Morthorst et al. [27] found lower mean DSHI-Y scores at follow-up in the ERITA group than in TAU, but the difference was not statistically significant. From the reported post-treatment means and standard deviations, we calculated Hedges’ g = −0.38 (95% CI −1.14 to 0.38). The estimate favored ERITA but was imprecise.

In Yuan et al.’s study [15], ACT plus routine psychological support produced greater reductions in Adolescent Non-Suicidal Self-Injury Questionnaire (ANSSIQ) behavior and function scores than routine support alone. Using T0–T2 change scores, the calculated effect for the ANSSIQ behavior questionnaire was Hedges’ g = −0.47 (95% CI −0.94 to 0.00), and the effect for the ANSSIQ function questionnaire was Hedges’ g = −1.14 (95% CI −1.64 to −0.64). These effects favor ACT, but their interpretation is limited by the retrospective non-randomized design.

### 3.4. Exploratory Meta-Analysis of NSSI Outcomes

**Figure 3:**
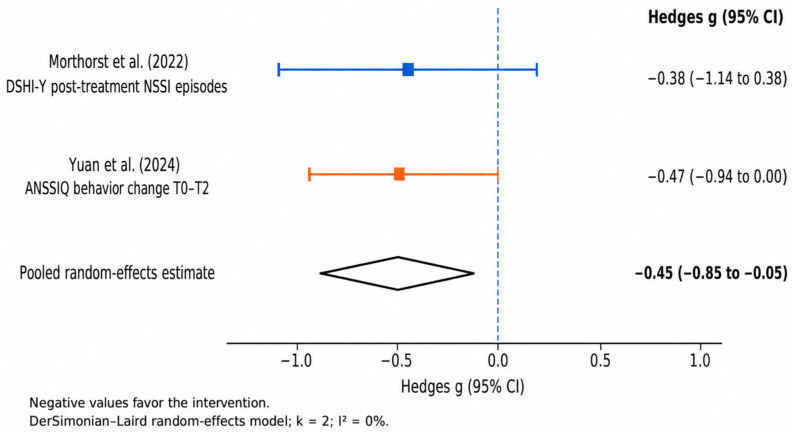
Exploratory random-effects synthesis of extractable continuous NSSI outcomes. Note. Study-level estimates are from Morthorst et al. [27] (blue) and Yuan et al. [15] (orange). Squares represent the study-specific effect estimates, and the corresponding horizontal lines represent their 95% CIs. The diamond represents the pooled random-effects estimate and its 95% CI, while the vertical dashed line marks the null value (Hedges’ g = 0). ANSSIQ = Adolescent Non-Suicidal Self-Injury Questionnaire; CI = confidence interval; DSHI-Y = Deliberate Self-Harm Inventory–Youth version; NSSI = non-suicidal self-injury; T0–T2 = change from baseline to final assessment; k = number of studies; I^2^ = percentage of total variation attributable to between-study heterogeneity. Effect sizes are Hedges’ g. With only two contributing studies, I^2^ is highly uncertain and should not be interpreted as evidence of homogeneity.

The primary exploratory SMD synthesis included one NSSI outcome per controlled study for which extractable continuous data were available: Morthorst et al. [27] DSHI-Y post-treatment NSSI episodes and Yuan et al. [15] ANSSIQ behavior change. The pooled random-effects estimate was Hedges’ g = −0.45 (95% CI −0.85 to −0.05; k = 2; I^2^ = 0%), with the direction of the estimate favoring the intervention conditions (Figure 3). This estimate is hypothesis-generating and inherently unstable because it combines only two studies that differed in intervention, study design, comparator, outcome measure, and assessment timing. It is therefore imprecise as an estimate of any generalizable treatment effect and should not be interpreted as evidence of efficacy. The observed I^2^ of 0% should therefore not be interpreted as evidence of substantive homogeneity.

A sensitivity synthesis additionally included the Yuan et al. [15] ANSSIQ function change score. The pooled estimate was larger, Hedges’ g = −0.70 (95% CI −1.18 to −0.21; k = 3; I^2^ = 55.8%), but this result was less conservative because two effects came from the same non-randomized study. Therefore, the primary synthesis was retained as the main quantitative estimate.

### 3.5. Effects on Emotion Regulation and ACT-Related Processes

Process outcomes were central to the rationale for these interventions. In the Swedish RCT, Bjureberg et al. [33] reported that improvement in emotion dysregulation mediated improvement in NSSI during treatment, supporting the hypothesized mechanism of IERITA. Because these process findings were reported as mediation results rather than as directly extractable post-treatment standardized mean differences comparable to the ACT studies, they were summarized narratively rather than included in the process SMD synthesis.

Yuan et al. [15] found a greater improvement in positive emotion regulation and a greater reduction in negative emotion regulation in the ACT group than in the control group. Falahati et al. [32] found post-treatment improvements in cognitive emotion regulation and emotional inhibition after ACT. Individual study-level effect estimates from the controlled studies are summarized in Table 3.

**Figure 4:**
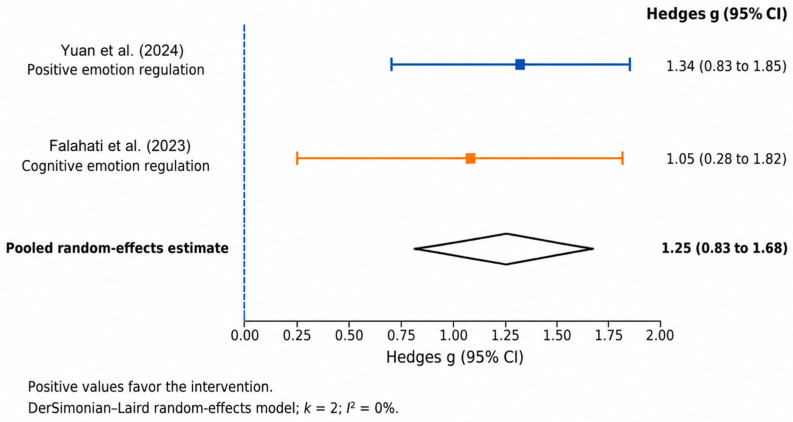
Exploratory random-effects synthesis of emotion regulation/process outcomes. Note. Study-level estimates are from Yuan et al. [15] (blue) and Falahati et al. [32] (orange). Squares represent the study-specific effect estimates, and the corresponding horizontal lines represent their 95% CIs. The diamond represents the pooled random-effects estimate and its 95% CI, while the vertical dashed line marks the null value (Hedges’ g = 0). CI = confidence interval; k = number of studies; I^2^ = percentage of total variation attributable to between-study heterogeneity. Effect sizes are Hedges’ g; positive values favor the intervention. With only two contributing studies, I^2^ is highly uncertain and should not be interpreted as evidence of homogeneity.

The exploratory random-effects synthesis included one process outcome from each of the two controlled ACT studies: positive emotion regulation change from Yuan et al. [15] and post-treatment cognitive emotion regulation from Falahati et al. [32]. The pooled estimate was Hedges’ g = 1.25 (95% CI 0.83 to 1.68; k = 2; I^2^ = 0%) (Figure 4). Although the numerical estimate was large, it should be regarded as hypothesis-generating rather than as a stable estimate of treatment effect. Its precision cannot be judged adequately from the nominal confidence interval alone, because the synthesis contained only two small studies and the estimated between-study variance is highly uncertain when k = 2. It was derived from only two small, methodologically limited studies with different designs, comparators, measures, and methods of calculating change. The observed I^2^ of 0% is highly imprecise with k = 2 and does not demonstrate homogeneity between the studies.

### 3.6. Feasibility, Acceptability, and Safety

Feasibility and acceptability outcomes were generally favorable, but safety reporting was incomplete and non-uniform. In the Morthorst et al. [27] feasibility RCT, 90% of participants completed post-treatment interviews and 87% completed at least six of eleven ERITA modules. Adverse effects were assessed with the Negative Effects Questionnaire, with no observed between-group difference (point estimate = 0; 95% CI −5 to 3; *p* = 0.97). The ERITA group showed fewer risk-related behaviors on the Borderline Symptom List supplement (point estimate = 1; 95% CI 0 to 3; *p* = 0.02), but the trial included only 30 participants and was not powered to establish safety. Two of the fifteen participants allocated to ERITA discontinued the intervention, one before starting and one after the introduction and first module; these discontinuations were not reported as resulting from clinical deterioration. Adolescents with imminent suicide risk requiring inpatient care were excluded.

In the larger IERITA RCT, 5 of 84 participants (6%) in the IERITA plus TAU group and 9 of 82 participants (11%) in the TAU-only group reported suicide attempts during the study period [33]. The trial excluded adolescents with immediate suicide risk and was not designed or powered to compare suicide attempt rates. These counts should therefore not be interpreted as evidence that IERITA prevents suicidal behavior or that its safety has been established for adolescents at acute risk.

In the online ERITA open pilot, severe suicidal ideation was an exclusion criterion, NSSI and suicidal ideation were monitored weekly, and an individualized crisis plan was established before treatment [25]. Eight of twenty-five adolescents reported at least one adverse event, most commonly increased distress (*n* = 7), followed by insufficient time for schoolwork or other obligations (*n* = 3) and feeling depressed when experiencing difficulty practicing the treatment skills (*n* = 1). Because the study was uncontrolled and involved a clinically selected sample, it cannot establish comparative safety.

In the face-to-face ERITA feasibility study, 2 of 17 participants discontinued after session 2 because of discomfort with or disinterest in the treatment or its format [35]; clinical deterioration was not given as the reason for discontinuation. The Yuan et al. [15] and Falahati et al. [32] studies did not report structured adverse event, suicidality, emergency service, hospitalization, treatment escalation, or clinically driven dropout outcomes. Across the included evidence base, emergency visits, hospitalizations, treatment escalation, individual-level worsening of NSSI, and discontinuation because of clinical decline were not reported consistently. Lack of reporting cannot be interpreted as absence of harm. Safety monitoring and reported safety-related outcomes are summarized in Table 4.

### 3.7. Publication and Availability Bias

Formal publication-bias testing was not performed because the number of studies contributing to each synthesis was far below the threshold at which funnel-plot asymmetry tests are informative. Both publication bias and availability bias remain plausible. Fifteen reports sought for retrieval could not be obtained in full text, including potentially relevant Chinese- and Persian-language records. Some international databases index titles or abstracts from regional journals without providing a reliable route to the full report, whereas regional platforms may contain additional bibliographic information or locally accessible full texts. The absence of direct searches in CNKI, Wanfang, SID, and Magiran therefore leaves uncertainty about whether relevant studies or usable reports were missed.

Selective publication is an additional concern in such a small and emerging literature. Studies reporting favorable or statistically significant findings may be more likely to reach full publication, whereas null, inconclusive, unfavorable, or prematurely discontinued studies may remain unpublished or be available only as dissertations, conference reports, registry entries, or regional publications. If such evidence is missing, the present synthesis may overestimate treatment benefits and underrepresent nonresponse, adverse outcomes, or lack of feasibility. The direction and magnitude of this potential bias cannot be quantified with the available number of studies.

## 4. Discussion

This systematic review and exploratory meta-analysis identified preliminary signals that primarily ACT, ERITA, and IERITA interventions warrant further investigation for adolescent NSSI, but it does not establish treatment efficacy. The strongest evidence concerns IERITA, which reduced clinician-rated NSSI frequency in a randomized clinical trial. The evidence for primarily ACT interventions is clinically encouraging but methodologically weaker, consisting mainly of retrospective or small quasi-experimental controlled studies. Accordingly, the pooled estimates do not establish treatment efficacy and should be understood as preliminary signals for future confirmatory trials rather than as a basis for firm comparative or clinical conclusions. Pooling was retained only as an exploratory descriptive summary because the studies addressed a common broad clinical question and reported conceptually aligned outcomes that could be expressed as standardized mean differences; it was not intended to imply intervention equivalence or a single common treatment effect. Evidence from the IERITA trial should therefore be interpreted as evidence for that specific multicomponent intervention and delivery format, not as confirmation of efficacy for ACT, ACT-informed interventions generally, or ERITA delivered in other formats. This interpretation is consistent with the GRADE assessment: certainty was moderate for the single masked clinician-rated IERITA outcome but very low for the pooled continuous NSSI, process, and feasibility evidence. The apparent consistency or magnitude of the pooled estimates should therefore not be interpreted as high-certainty evidence.

Clinical and methodological heterogeneity materially limits both the validity and the interpretability of the pooled estimates. The primary continuous NSSI synthesis combined a small randomized ERITA feasibility trial with a retrospective non-randomized primarily ACT study. These studies differed not only in intervention content but also in allocation method, comparator, outcome measurement, assessment timing, and susceptibility to confounding and selection bias. The process–outcome synthesis included two primarily ACT studies, but both were methodologically limited and assessed different emotion-regulation constructs under different comparator conditions. Consequently, the summary estimates may combine genuine intervention effects with differences arising from study design, comparator strength, therapist contact, treatment intensity, parental involvement, delivery format, selection processes, and outcome measurement. Expressing the outcomes as standardized mean differences places them on a common numerical scale but does not establish clinical or methodological equivalence. Similarly, a random-effects model allows underlying effects to vary but cannot correct confounding, selection bias, measurement bias, weak comparator conditions, or conceptual non-equivalence between interventions. With only two studies in each principal synthesis, the between-study variance and I^2^ are also too imprecise to determine whether the apparent numerical consistency reflects genuine similarity. Combining the studies was therefore considered acceptable only for an exploratory descriptive summary of direction and approximate magnitude. The pooled estimates should not be interpreted as valid estimates of a common intervention effect, and the individual-study and intervention-specific findings remain the primary basis for interpretation.

The apparently large pooled process estimate (Hedges’ g = 1.25) warrants particular caution. It was derived from only two small controlled ACT studies: one retrospective non-randomized study and one semi-experimental study with unclear allocation procedures. Neither study used concealed randomization or a credible attention-matched active comparator, and the process constructs, measures, and analytic approaches were not equivalent. Confounding, selection effects, expectancy, differential therapeutic attention, and outcome measurement bias may therefore have contributed to the observed magnitude. The estimate should be interpreted as a preliminary signal that emotion regulation processes warrant further testing, rather than as evidence of a large, reproducible mechanism effect. Future trials should use adequately powered prospective randomization, concealed allocation, prespecified process outcomes, blinded outcome assessment where feasible, and active comparators matched for therapist contact, treatment intensity, and expectancy.

These findings are theoretically coherent. NSSI is often maintained by emotion regulation and experiential avoidance functions [13,14]. Primarily ACT interventions directly target these mechanisms through acceptance, defusion, values clarification, and committed action, whereas ERITA and IERITA principally target emotion dysregulation while incorporating selected acceptance-related and ACT-consistent components [16]. In this sense, the aim of treatment is not simply to suppress self-injury urges, but to change the adolescent’s relationship with distress and to increase behavioral flexibility when urges arise.

The available follow-up evidence is insufficient to establish the durability of either the clinical or process-related findings. Controlled evidence extended only to three months after treatment in the IERITA trial, whereas maintenance at six months was reported only in a small uncontrolled ERITA pilot. The remaining studies primarily assessed outcomes at treatment completion or over similarly short intervals. These data cannot determine whether reductions in NSSI persist, whether relapse or delayed recurrence occurs, or whether improvements in emotion regulation and psychological flexibility translate into sustained gains in functioning, reduced service use, or lower longer-term suicidal risk. Future trials should include multiple prespecified follow-up assessments extending beyond six months and, where feasible, to 12 months or longer.

IERITA is clinically relevant because it delivers a structured, therapist-guided intervention through an internet format and includes parent modules. This format may offer practical advantages in accessibility and scalability, but internet delivery should not be equated with low clinical risk or universal suitability. The principal digital studies involved therapist guidance, concurrent treatment as usual, parent involvement or explicit crisis procedures, and excluded adolescents with immediate, imminent, or severe suicide risk. Their findings therefore do not generalize to unsupported self-guided digital treatment or to adolescents requiring urgent or inpatient care. At most, therapist-guided IERITA may currently be considered a promising adjunctive or stepped-care option for carefully selected adolescents whose clinical risk can be managed safely in outpatient care. This interpretation remains provisional because no included study directly compared IERITA with DBT-A, mentalization-based treatment for adolescents (MBT-A), or specialist multimodal care, and comparative effectiveness, resource use, safety, treatment sequencing, and criteria for patient selection remain uncertain. Current evidence does not support replacing DBT-A or other established intensive treatments with IERITA, particularly for adolescents at high risk. Adolescents with acute or escalating suicide risk, repeated suicidal behavior, severe or complex psychiatric comorbidity, complex trauma, marked interpersonal instability, or substantial family dysfunction require comprehensive specialist assessment, active risk management, and clear escalation pathways. Because high-risk adolescents were excluded from key trials and safety outcomes were inconsistently reported, the absence of an observed excess of serious events cannot be interpreted as evidence of safety.

The review also highlights an important methodological tension. If the topic is framed narrowly as primarily ACT for adolescent NSSI, the evidence base is small and weak. If it is framed more broadly to include ACT-informed, acceptance-based, ERITA, and IERITA interventions, the evidence base becomes more clinically meaningful, but effects cannot be attributed uniquely to ACT. The present title deliberately uses the broader formulation to reflect the actual state of the literature.

The distinction between primary adolescent NSSI evidence and contextual ACT evidence is also important. The ACT-DEA study by Na et al. [34] is relevant to the theoretical model because it targets destructive experiential avoidance and includes self-harm behavior, but it is not a clean adolescent NSSI efficacy study. Excluding it from the main synthesis therefore strengthens the methodological defensibility of the review while preserving its conceptual relevance.

Several limitations should be considered when interpreting these findings. First, the search was not exhaustive. Although it included PubMed, Scopus, Web of Science, and EBSCO, it did not comprehensively cover Embase, CENTRAL, trial registries, dissertation databases, or regional databases. In particular, direct searches of CNKI and Wanfang for Chinese literature and SID and Magiran for Persian literature were not performed. Relevant regional, unpublished, registered, or dissertation evidence may therefore have been missed. Second, 15 reports sought for retrieval could not be obtained in full text, including potentially relevant Chinese- and Persian-language records, and consequently remained unassessed for eligibility. This creates availability bias in addition to possible identification bias, because international databases may index titles or abstracts without providing access to the underlying report, while regional platforms may contain further records or accessible local full texts. The included studies should therefore not be regarded as a complete representation of all potentially eligible evidence. In a review containing only six primary studies, even one or two additional eligible reports could materially alter the exploratory pooled estimates and the relative conclusions concerning primarily ACT, ERITA, and IERITA interventions. Future updates should incorporate targeted regional and gray literature searches, supported where necessary by local-language searching, author contact, and interlibrary retrieval. Selective publication of positive findings is also plausible and may have inflated the apparent magnitude and consistency of benefit, while null, unfavorable, or safety-related findings may be underrepresented. This possibility could not be examined formally because each synthesis contained only two or three effects. Third, only a very small number of controlled studies had extractable quantitative data, and the pooled estimates were based on very few effects. Fourth, substantial clinical and methodological heterogeneity limits the interpretability of the pooled estimates. The studies differed in intervention content, delivery format, therapist guidance, parental involvement, treatment intensity, comparator, study design, outcome measurement, and assessment timing. The pooled effects may therefore combine intervention-specific effects with differences arising from delivery conditions, comparator strength, and risk of bias, and cannot be interpreted as a single underlying ACT effect. Fifth, the primarily ACT evidence was limited by non-randomized or small quasi-experimental designs. Sixth, follow-up periods were short. Controlled evidence extended only to three months after treatment, and the only six-month finding came from a small uncontrolled ERITA pilot. Long-term durability, relapse, delayed recurrence, sustained process change, and longer-term functional and safety outcomes therefore remain unknown. Most samples were also predominantly female, limiting generalizability. Seventh, because the OSF record was registered retrospectively, it did not prospectively constrain all eligibility, extraction, risk of bias, and quantitative analysis decisions. Although the timing and rationale of the principal post hoc adaptations are now reported explicitly, selective analytic flexibility cannot be excluded. Eighth, safety reporting was inconsistent and generally inadequate. Suicidal ideation and attempts, emergency or crisis service use, hospitalization, treatment escalation, clinically meaningful worsening of NSSI, and discontinuation because of clinical decline were not assessed or reported consistently. Several studies excluded adolescents with immediate, imminent, or severe suicide risk, and the available trials were not powered to detect differences in rare but clinically serious events. Consequently, this review cannot establish the safety of primarily ACT, ERITA, or IERITA interventions, particularly for adolescents with repeated suicidal behavior, acute risk, or complex comorbidity. For these reasons, the quantitative findings should be interpreted as hypothesis-generating rather than definitive.

## 5. Conclusions

Primarily ACT, ERITA, and IERITA interventions warrant further investigation for adolescent NSSI, particularly in relation to emotion regulation and psychological flexibility. The available evidence is insufficient to establish treatment efficacy. Using GRADE, certainty was moderate for the single masked clinician-rated IERITA outcome and very low for the pooled continuous NSSI, process, and feasibility evidence. The pooled estimates are post hoc, exploratory, hypothesis-generating, and imprecise and should be understood only as summaries of preliminary signals across conceptually related outcomes. They do not represent a single common treatment effect and should not be generalized across primarily ACT, ERITA, IERITA, internet-delivered, face-to-face, or parent-inclusive interventions, because the relative contribution of these treatment and delivery characteristics remains unknown. The strongest single-study evidence concerns therapist-guided IERITA, while evidence for primarily ACT interventions remains preliminary and is derived from methodologically limited studies. Larger preregistered randomized controlled trials with adequately generated randomization sequences, concealed allocation, and prospectively specified analyses are needed to evaluate each intervention category separately against credible active comparators. Comparators should be matched, where possible, for therapist contact, treatment intensity, and expectancy. Trials should also use standardized NSSI outcomes, multiple prespecified follow-up assessments extending beyond six months, intention-to-treat analyses, and prespecified mediation models testing psychological flexibility, experiential avoidance, cognitive fusion, emotional inhibition, and emotion regulation.

Therapist-guided IERITA may have a future role as an adjunctive or stepped-care option for carefully selected adolescents, but this clinical position has not yet been established. Current evidence does not support replacing DBT-A, MBT-A, or specialist multimodal care with IERITA, particularly for adolescents with acute or escalating suicide risk, repeated suicidal behavior, or severe and complex psychiatric comorbidity. Safety remains uncertain because serious clinical events and service-use outcomes were reported inconsistently and key trials excluded adolescents with an immediate or imminent suicide risk. Until adequately powered safety and comparative-effectiveness evidence is available, IERITA should be delivered only within a clinical framework that includes comprehensive assessment, ongoing suicide-risk monitoring, crisis planning, access to urgent care, and explicit criteria for treatment escalation. Evidence is also insufficient to select IERITA, ERITA, or primarily ACT interventions solely on the assumption that NSSI is maintained by experiential avoidance or emotion dysregulation. Future comparative trials should examine treatment sequencing, escalation criteria, safety, acceptability, resource use, and moderators of response.

## Figures and Tables

**Table 1:** Comparative summary of intervention characteristics, methodological appraisal, and principal findings.

Study and Sample	Intervention Characteristics	Design and Comparator	Overall Methodological Appraisal	Principal Findings
Yuan et al. [15]; China; 72 adolescents aged 13–18 years with DSM-5 NSSI	Primarily ACT plus routine psychological support; nine 60 min group sessions delivered over six weeks	Retrospective controlled study; routine psychological support alone	Serious risk of bias because allocation was non-randomized and based on management method; residual confounding, selection effects, and unblinded self-reported outcomes limit causal interpretation	ACT was associated with greater reductions in ANSSIQ behavior and function scores and greater improvement in positive and negative emotion regulation than routine support
Bjureberg et al. [25]; Sweden; 25 adolescents aged 13–17 years with NSSI disorder	Online ERITA; 11 adolescent modules plus a parallel therapist-guided online parent program	Uncontrolled open pilot; no comparator	Critical risk of bias for causal efficacy because pre–post change could not be separated from time, regression to the mean, concurrent treatment, or expectancy effects	Treatment was feasible and acceptable; past-month NSSI frequency decreased by 55% at post-treatment, with further improvement at three months and maintenance at six months
Morthorst et al. [27]; Denmark; 30 adolescents aged 13–17 years with recurrent NSSI	Internet-based ERITA plus TAU; 11-week intervention	Randomized feasibility trial; TAU comparator	Some concerns because the trial was small, not powered for efficacy, allocation concealment details were limited, and some outcome data were missing	Completion and intervention compliance were high; the NSSI estimate favored ERITA, but the between-group difference was not statistically significant and the confidence interval was wide
Falahati et al. [32]; Iran; 30 girls aged 11–13 years with NSSI	Primarily ACT; eight weekly 90 min sessions	Semi-experimental controlled study; no-training control	Serious risk of bias because allocation was insufficiently described, no concealed randomization or active comparator was used, and outcomes were assessed without blinding	Cognitive emotion regulation and emotional inhibition improved more in the ACT group; no directly extractable NSSI-frequency outcome was reported
Bjureberg et al. [33]; Sweden; 166 adolescents aged 13–17 years with NSSI disorder	Therapist-guided IERITA plus TAU; 12 weeks; adolescent and parent modules	Three-site, single-masked randomized clinical trial; TAU comparator	Strongest design in the evidence base, although some concerns remained because treatment delivery could not be blinded and 154 of 166 randomized participants contributed to the post-treatment analysis	Masked assessor-rated NSSI frequency was lower with IERITA plus TAU than with TAU alone, IRR = 0.34 (95% CI 0.20 to 0.57), with maintenance at three-month follow-up
Bjureberg et al. [35]; Sweden; 17 girls aged 13–17 years with NSSI disorder	Face-to-face ERITA for adolescents plus a parallel online parent program	Uncontrolled open feasibility study; no comparator	Critical risk of bias for causal efficacy because the sample was very small and no comparator or blinded assessment was available	Treatment credibility, alliance, attendance, and completion were acceptable; pre–post improvements were reported in NSSI frequency, NSSI versatility, emotion regulation difficulties, self-destructive behaviors, and global functioning

Note. ACT = acceptance and commitment therapy; ANSSIQ = Adolescent Non-Suicidal Self-Injury Questionnaire; DSM-5 = Diagnostic and Statistical Manual of Mental Disorders, Fifth Edition; ERITA = emotion regulation individual therapy for adolescents; IERITA = internet-delivered emotion regulation individual therapy for adolescents; IRR = incidence rate ratio; NSSI = non-suicidal self-injury; TAU = treatment as usual. The methodological appraisals provide concise study-level summaries; full domain-level risk-of-bias judgments and justifications are presented in Section 3.2. Primarily ACT, ACT-informed, acceptance-based, ERITA, and IERITA were treated as distinct intervention classifications. No included primary study met the criteria for a standalone ACT-informed or acceptance-based intervention. ERITA and IERITA were treated as related but distinct multicomponent emotion regulation interventions containing selected acceptance-related and ACT-consistent elements; neither was treated as a complete ACT protocol or as direct evidence of ACT-specific efficacy. Na et al. [34] was not counted as a primary adolescent NSSI intervention study because it included a mixed adolescent/young-adult sample and targeted mixed destructive experiential avoidance behaviors.

**Table 2:** Domain-level risk-of-bias judgments and brief study-specific justifications.

Study	Randomization/Confounding	Selection/Allocation	Missing Data	Outcome Measurement	Selective Reporting	Overall
Yuan et al. [15]	Serious. Retrospective non-randomized allocation based on management method; residual confounding is likely.	Serious. No concealed random allocation; assignment may have reflected clinical or service-related factors.	Moderate. Attrition and the handling of missing outcome data were not reported in sufficient detail.	Serious. Outcomes were self-reported and assessed without blinding, making the findings vulnerable to differential measurement and expectancy effects.	Some concerns. No prospective protocol or statistical analysis plan was available, and several outcomes were reported.	Serious. Confounding and selection substantially limit causal interpretation.
Bjureberg et al. [25]	Critical. Single-arm design without a comparator.	Critical. Selected pilot sample and no allocation process.	Some concerns. Small sample and limited information on the handling of missing follow-up data.	Some concerns. Outcomes were primarily self-reported in an unblinded study.	Some concerns. Pilot study with limited prespecification of efficacy analyses.	Critical for causal efficacy. Intervention effects cannot be separated from time, regression to the mean, concurrent care, or expectancy.
Morthorst et al. [27]	Low. Randomized allocation was reported.	Some concerns. Allocation-concealment details were limited.	Some concerns. Post-treatment DSHI-Y data were available for 13 of 15 and 14 of 15 participants.	Low. A standardized NSSI measure was assessed at a defined follow-up, with no evidence of differential measurement between groups.	Low. No clear evidence of selective omission; the efficacy analyses were explicitly described as exploratory.	Some concerns. Limited reporting of allocation concealment and some missing outcome data create some concerns about bias.
Falahati et al. [32]	Serious. The semi-experimental design and insufficiently described allocation method leave substantial potential for confounding.	Serious. There was no concealed randomization, and baseline selection differences cannot be excluded.	Some concerns. Attrition and missing-data handling were insufficiently described.	Serious. Process outcomes were assessed without blinding, and no direct extractable NSSI-frequency outcome was reported.	Some concerns. No accessible protocol or prespecified statistical analysis plan was available.	Serious. Allocation and measurement limitations preclude strong causal inference.
Bjureberg et al. [33]	Low. Random assignment was used in a controlled clinical trial.	Some concerns. Participants and therapists could not be blinded, and deviations from assigned care cannot be excluded completely.	Some concerns. A total of 154 of 166 randomized participants contributed to the post-treatment analysis.	Low. The primary NSSI outcome was assessed by masked clinicians using a predefined measure.	Low. The trial was registered, and the primary outcome and model-based analysis were clearly reported.	Some concerns. Some outcome data were missing and participants and treatment providers could not be blinded, although masked assessment of the primary outcome reduced concern about measurement bias.
Bjureberg et al. [35]	Critical. Single-arm design without a comparator.	Critical. Selected feasibility sample and no allocation process.	Some concerns. The sample was very small, with limited detail on missing-data handling.	Some concerns. Outcomes were primarily self-reported and assessed without blinding.	Some concerns. Open feasibility study with limited prespecification of efficacy analyses.	Critical for causal efficacy. Pre–post change cannot be attributed specifically to the intervention.

Note. The categories summarize the likely direction and severity of bias rather than constituting a numerical score. Open single-arm studies were judged at critical risk of bias for causal efficacy because no comparator was available.

**Table 3:** Quantitative findings from controlled studies.

Study	Outcome	Data Used	Effect Estimate	Interpretation
Yuan et al. [15]	ANSSIQ behavior questionnaire	T0–T2 change score	Hedges’ g = −0.47 (95% CI −0.94 to 0.00)	Direction favors ACT; small-to-moderate but borderline/imprecise
	ANSSIQ function questionnaire	T0–T2 change score	Hedges’ g = −1.14 (95% CI −1.64 to −0.64)	Large effect favoring ACT; sensitivity outcome from the same non-randomized study
	Positive emotion regulation	T0–T2 change score	Hedges’ g = 1.34 (95% CI 0.83 to 1.85)	Numerically large process estimate; retrospective non-randomized design and absence of a matched active comparator limit causal interpretation
Morthorst et al. [27]	DSHI-Y NSSI episodes	Post-treatment mean/SD	Hedges’ g = −0.38 (95% CI −1.14 to 0.38)	Direction favors ERITA; feasibility trial, imprecise and not statistically significant
Falahati et al. [32]	Cognitive emotion regulation	Post-treatment mean/SD	Hedges’ g = 1.05 (95% CI 0.28 to 1.82)	Numerically large process estimate; small semi-experimental study with unclear allocation and no active comparator
Bjureberg et al. [33]	Masked clinician-rated NSSI frequency	Reported model-based count outcome	IRR = 0.34 (95% CI 0.20 to 0.57)	Favors IERITA + TAU; reported separately because it is not an SMD

Note. Negative values indicate lower NSSI severity/frequency in the intervention group; positive values indicate improvement in process outcomes. ACT = acceptance and commitment therapy; ANSSIQ = Adolescent Non-Suicidal Self-Injury Questionnaire; CI = confidence interval; DSHI-Y = Deliberate Self-Harm Inventory–Youth version; ERITA = Emotion regulation individual therapy for adolescents; IERITA = internet-delivered emotion regulation individual therapy for adolescents; IRR = incidence rate ratio; NSSI = non-suicidal self-injury; SMD = standardized mean difference; TAU = treatment as usual; T0–T2 = change from baseline to final assessment. Except for the Bjureberg et al. [33] IRR, effect sizes were calculated from aggregate data. The two Yuan et al. [15] ANSSIQ estimates are non-independent. Full numerical inputs and calculation assumptions are reported in Supplementary Table S2.

**Table 4:** Safety monitoring and reported safety-related outcomes in the included primary studies.

Study	Safety monitoring or Eligibility Restrictions	Reported Safety-Related Findings	Interpretation
Yuan et al. [15]	No structured safety-monitoring procedure described	No explicit adverse event, suicidality, emergency visit, hospitalization, treatment-escalation, or clinically driven dropout data reported	Safety cannot be inferred from non-reporting
Bjureberg et al. [25]	Severe suicidal ideation excluded; weekly monitoring of NSSI and suicidal ideation; individualized crisis plan	Eight of twenty-five participants reported at least one adverse event: increased distress (*n* = 7), time burden (*n* = 3), and depressed mood when struggling with treatment skills (*n* = 1)	Uncontrolled, clinically selected sample; no comparative safety inference
Morthorst et al. [27]	Imminent suicidal risk requiring inpatient care excluded; NEQ and BSL-supplement administered	No between-group difference in NEQ scores; fewer BSL risk-related behaviors in ERITA; 2 of 15 ERITA participants discontinued, not reportedly because of clinical decline	Small feasibility trial not powered to establish safety
Falahati et al. [32]	No structured safety monitoring procedure described	No explicit safety, suicidality, service use, or clinically driven dropout outcomes reported	Safety cannot be inferred from non-reporting
Bjureberg et al. [33]	Immediate suicide risk and urgent-treatment needs were exclusion criteria; adverse events and suicidal behavior were assessed during follow-up	Suicide attempts were reported by 5/84 participants in IERITA + TAU and 9/82 participants in TAU; emergency visits, hospitalizations, and treatment escalation were not reported systematically	The trial was not designed or powered to compare suicidal behavior; the counts do not establish a protective effect or definitive safety
Bjureberg et al. [35]	Participants remained in community psychiatric care	Two of seventeen discontinued because of discomfort with or disinterest in treatment or format; no systematic emergency, hospitalization, or suicidal-event results	Uncontrolled study; safety not established

Note. BSL = Borderline Symptom List; ERITA = emotion regulation individual therapy for adolescents; IERITA = internet-delivered emotion regulation individual therapy for adolescents; NEQ = Negative Effects Questionnaire; NSSI = non-suicidal self-injury; TAU = treatment as usual. “Not reported” does not mean that no event occurred.

## Data Availability

No new primary data were generated in this study. The data analyzed in this systematic review and exploratory meta-analysis were extracted from previously published studies cited in the article. The aggregate data and effect estimates supporting the findings are reported within the manuscript and its tables/figures. Further details are available from the corresponding author upon reasonable request.

## Supplementary Materials

The following supporting information can be downloaded at https://www.mdpi.com/article/10.3390/children13070972/s1, Table S1. PRISMA 2020 Checklist. Table S2. Study-level numerical inputs and effect-size derivations for the quantitative syntheses. Table S3. Grading of Recommendations Assessment, Development and Evaluation (GRADE) certainty ratings for the principal outcome domains.

## Abbreviations

The following abbreviations are used in this manuscript:

ACT	Acceptance and commitment therapy
ACT-DEA	Acceptance and commitment therapy for destructive experiential avoidance
ANSSIQ	Adolescent Non-Suicidal Self-Injury Questionnaire
BSL	Borderline Symptom List
CENTRAL	Cochrane Central Register of Controlled Trials
CI	Confidence interval
DBT-A	Dialectical behavior therapy for adolescents
DSHI-Y	Deliberate Self-Harm Inventory–Youth version
DSM-5	Diagnostic and Statistical Manual of Mental Disorders, Fifth Edition
ERITA	Emotion regulation individual therapy for adolescents
GRADE	Grading of Recommendations Assessment, Development and Evaluation
IERITA	Internet-delivered emotion regulation individual therapy for adolescents
IRR	Incidence rate ratio
MBT-A	Mentalization-based treatment for adolescents
NEQ	Negative Effects Questionnaire
NSSI	Non-suicidal self-injury
OSF	Open Science Framework
PRISMA	Preferred Reporting Items for Systematic Reviews and Meta-Analyses
RCT	Randomized controlled trial
RoB 2	Revised Cochrane risk-of-bias tool for randomized trials
ROBINS-I	Risk of Bias in Non-randomized Studies of Interventions
SMD	Standardized mean difference
TAU	Treatment as usual
