# Supplementary material for: Acceptance-Based and ACT-Informed Interventions for Non-Suicidal Self-Injury in Adolescents: A Systematic Review and Exploratory Meta-Analysis
Source: Children (Basel). 2026 Jul 22;13(7):972. doi: 10.3390/children13070972 (PMC13406886; doi:10.3390/children13070972)
Supplement: Supplementary file 1 [file children-13-00972-s001.zip › Table S1.pdf]

Supplementary Table S1. PRISMA 2020 Checklist

Manuscript: Acceptance-based and ACT-informed interventions for non-suicidal self-injury in adolescents: a systematic review and exploratory meta-analysis

Section and Topic	Item #	Checklist item	Location where item is reported
**TITLE**			
Title	1	Identify the report as a systematic review.	Title, lines 1-4.
**ABSTRACT**			
Abstract	2	See the PRISMA 2020 for Abstracts checklist.	Abstract, lines 25-67.
**INTRODUCTION**			
Rationale	3	Describe the rationale for the review in the context of existing knowledge.	Introduction, lines 71-113.
Objectives	4	Provide an explicit statement of the objective(s) or question(s) the review addresses.	Introduction, lines 114-119.
**METHODS**			
Eligibility criteria	5	Specify the inclusion and exclusion criteria for the review and how studies were grouped for the syntheses.	Methods 2.1, lines 211-231; Methods 2.2, lines 262-287; Methods 2.4, lines 312-317 and 360-378.
Information sources	6	Specify all databases, registers, websites, organisations, reference lists and other sources searched or consulted to identify studies. Specify the date when each source was last searched or consulted.	Methods 2.1, lines 155-164; Methods 2.2, lines 239-245.
Search strategy	7	Present the full search strategies for all databases, registers and websites, including any filters and limits used.	Methods 2.1, lines 155-210 (date, limits, and full PubMed, Scopus, Web of Science, and EBSCO strategies).
Selection process	8	Specify the methods used to decide whether a study met the inclusion criteria of the review, including how many reviewers screened each record and each report retrieved, whether they worked independently, and if applicable, details of automation tools used in the process.	Methods 2.2, lines 233-245; Figure 1, lines 417-419 (no automation tools used).
Data collection process	9	Specify the methods used to collect data from reports, including how many reviewers collected data from each report, whether they worked independently, any processes for obtaining or confirming data from study investigators, and if applicable, details of automation tools used in the process.	Methods 2.2, lines 246-261.
Data items	10a	List and define all outcomes for which data were sought. Specify whether all results that were compatible with each outcome domain in each study were sought (e.g. for all measures, time points, analyses), and if not, the methods used to decide which results to collect.	Methods 2.2, lines 246-261; Methods 2.4, lines 312-317 and 366-373.
	10b	List and define all other variables for which data were sought (e.g. participant and intervention characteristics, funding sources). Describe any assumptions made about any missing or unclear information.	Methods 2.2, lines 246-261; missing safety outcomes were recorded as "not reported" (lines 253-254).
Study risk of bias assessment	11	Specify the methods used to assess risk of bias in the included studies, including details of the tool(s) used, how many reviewers assessed each study and whether they worked independently, and if applicable, details of automation tools used in the process.	Methods 2.3, lines 289-300; Results 3.2, lines 509-538; Table 2.
Effect measures	12	Specify for each outcome the effect measure(s) (e.g. risk ratio, mean difference) used in the synthesis or presentation of results.	Methods 2.4, lines 312-359 and 393-395.
Synthesis methods	13a	Describe the processes used to decide which studies were eligible for each synthesis (e.g. tabulating the study intervention characteristics and comparing against the planned groups for each synthesis (item #5)).	Methods 2.1, lines 211-231; Methods 2.2, lines 262-287; Methods 2.4, lines 360-378; Section 2.5, lines 407-415.
	13b	Describe any methods required to prepare the data for presentation or synthesis, such as handling of missing summary statistics, or data conversions.	Methods 2.2, lines 255-261; Methods 2.4, lines 318-359; Supplementary Table S2.
	13c	Describe any methods used to tabulate or visually display results of individual studies and syntheses.	Results 3.1–3.6, lines 422–674; Tables 1–4; Figures 1–4; Supplementary Tables S2–S3. Study characteristics, risk-of-bias judgments, and safety findings were presented in structured tables, and individual and pooled quantitative estimates were displayed in forest plots.
	13d	Describe any methods used to synthesize results and provide a rationale for the choice(s). If meta-analysis was performed, describe the model(s), method(s) to identify the presence and extent of statistical heterogeneity, and software package(s) used.	Methods 2.4, lines 360–396 (rationale, effect-size calculations, IBM SPSS Statistics version 31.0, random-effects model, DerSimonian–Laird estimator, I^2, and treatment of heterogeneity).
	13e	Describe any methods used to explore possible causes of heterogeneity among study results (e.g. subgroup analysis, meta-regression).	Not applicable: no subgroup analysis or meta-regression was conducted because each synthesis contained only two or three effects; heterogeneity was considered qualitatively in Methods 2.4, lines 379-392, and Discussion, lines 715-735.
	13f	Describe any sensitivity analyses conducted to assess robustness of the synthesized results.	Methods 2.1, lines 142-148; Results 3.4, lines 583-587.
Reporting bias assessment	14	Describe any methods used to assess risk of bias due to missing results in a synthesis (arising from reporting biases).	Methods 2.3, lines 301–310; Methods 2.4, lines 388–392; Results 3.7, lines 675–693; Supplementary Table S3.
Certainty assessment	15	Describe any methods used to assess certainty (or confidence) in the body of evidence for an outcome.	Methods 2.3, lines 301-310; Supplementary Table S3.
**RESULTS**			
Study selection	16a	Describe the results of the search and selection process, from the number of records identified in the search to the number of studies included in the review, ideally using a flow diagram.	Section 2.5, lines 397-416; Figure 1, lines 417-419.
	16b	Cite studies that might appear to meet the inclusion criteria, but which were excluded, and explain why they were excluded.	Section 2.5, lines 400–406, and Figure 1 report aggregate categories and numbers of full-text exclusions. Na et al. [34] is discussed as contextual rather than primary evidence in Results 3.1, lines 488–494. A report-level list of excluded full-text studies with individual reasons is not available.
Study characteristics	17	Cite each included study and present its characteristics.	Results 3.1, lines 422-508; Table 1.
Risk of bias in studies	18	Present assessments of risk of bias for each included study.	Results 3.2, lines 509-538; Table 2.
Results of individual studies	19	For all outcomes, present, for each study: (a) summary statistics for each group (where appropriate) and (b) an effect estimate and its precision (e.g. confidence/credible interval), ideally using structured tables or plots.	Results 3.3-3.6, lines 548-674; Tables 3-4; Figures 2-4; Supplementary Table S2.
Results of syntheses	20a	For each synthesis, briefly summarise the characteristics and risk of bias among contributing studies.	Results 3.2, lines 509-547; Results 3.4-3.5, lines 573-628; Tables 1-3.

Supplementary Table S1. PRISMA 2020 Checklist

Section and Topic	Item #	Checklist item	Location where item is reported
	20b	Present results of all statistical syntheses conducted. If meta-analysis was done, present for each the summary estimate and its precision (e.g. confidence/credible interval) and measures of statistical heterogeneity. If comparing groups, describe the direction of the effect.	Results 3.3-3.5, lines 548-634; Table 3; Figures 2-4.
	20c	Present results of all investigations of possible causes of heterogeneity among study results.	Not applicable: no formal subgroup or meta-regression analysis was possible; heterogeneity is interpreted in Results 3.4-3.5, lines 578-582 and 622-628, and Discussion, lines 715-735.
	20d	Present results of all sensitivity analyses conducted to assess the robustness of the synthesized results.	Results 3.4, lines 583-587.
Reporting biases	21	Present assessments of risk of bias due to missing results (arising from reporting biases) for each synthesis assessed.	Results 3.7, lines 675-693.
Certainty of evidence	22	Present assessments of certainty (or confidence) in the body of evidence for each outcome assessed.	Results 3.2, lines 539-547; Supplementary Table S3.
**DISCUSSION**			
Discussion	23a	Provide a general interpretation of the results in the context of other evidence.	Discussion, lines 695-757.
	23b	Discuss any limitations of the evidence included in the review.	Discussion, lines 715-749, 758-767, and 823-847.
	23c	Discuss any limitations of the review processes used.	Discussion, lines 802-822 and 836-839.
	23d	Discuss implications of the results for practice, policy, and future research.	Discussion, lines 768-789; Conclusions, lines 851-886.
**OTHER INFORMATION**			
Registration and protocol	24a	Provide registration information for the review, including register name and registration number, or state that the review was not registered.	Methods 2.1, lines 122-126: Open Science Framework retrospective registration, DOI 10.17605/OSF.IO/2AK69.
	24b	Indicate where the review protocol can be accessed, or state that a protocol was not prepared.	Methods 2.1, lines 124-137: no separate prospectively prepared protocol; the OSF record is retrospective.
	24c	Describe and explain any amendments to information provided at registration or in the protocol.	Methods 2.1, lines 127-153: timing and post hoc methodological adaptations, including eligibility clarification, intervention classification, outcome selection, risk-of-bias and certainty methods, separate IRR reporting, and sensitivity analysis.
Support	25	Describe sources of financial or non-financial support for the review, and the role of the funders or sponsors in the review.	Funding statement, line 894.
Competing interests	26	Declare any competing interests of review authors.	Conflicts of Interest statement, line 903.
Availability of data, code and other materials	27	Report which of the following are publicly available and where they can be found: template data collection forms; data extracted from included studies; data used for all analyses; analytic code; any other materials used in the review.	Supplementary Tables S1–S3; Data Availability Statement, lines 897–901. Study-level numerical inputs and effect-size derivations are provided in Supplementary Table S2, and GRADE certainty judgments are provided in Supplementary Table S3. No analytic code or template data-collection form is publicly available.

From: Page MJ, McKenzie JE, Bossuyt PM, Boutron I, Hoffmann TC, Mulrow CD, et al. The PRISMA 2020 statement: an updated guideline for reporting systematic reviews. BMJ 2021;372:n71. doi: 10.1136/bmj.n71.
This work is licensed under CC BY 4.0. To view a copy of this license, visit https://creativecommons.org/licenses/by/4.0/
