# Supplementary material for: Acceptance-Based and ACT-Informed Interventions for Non-Suicidal Self-Injury in Adolescents: A Systematic Review and Exploratory Meta-Analysis
Source: Children (Basel). 2026 Jul 22;13(7):972. doi: 10.3390/children13070972 (PMC13406886; doi:10.3390/children13070972)
Supplement: Supplementary file 1 [file children-13-00972-s001.zip › Table S2.pdf]

Supplementary Table S2. Study-level numerical inputs and effect-size derivations for the quantitative syntheses.

Study and outcome	Role in synthesis	Intervention	Comparator	Time point and data used	Direction of scoring	Effect estimate
Yuan et al. [15], ANSSIQ behavior questionnaire	Primary NSSI synthesis	ACT + routine support: (n = 36), mean change = −11.72, SD = 8.92	Routine support: (n = 36), mean change = −7.22, SD = 9.84	T2−T0 change score; T2 at week 12	Higher raw scores indicate more severe NSSI; a more negative change indicates greater improvement	Hedges g = −0.47 (95% CI −0.94 to 0.00)
Yuan et al. [15], ANSSIQ function questionnaire	NSSI sensitivity synthesis only	ACT + routine support: (n = 36), mean change = −28.86, SD = 9.83	Routine support: (n = 36), mean change = −17.50, SD = 9.92	T2−T0 change score; T2 at week 12	Higher raw scores indicate greater NSSI functional reinforcement; a more negative change indicates improvement	Hedges g = −1.14 (95% CI −1.64 to −0.64)
Yuan et al. [15], positive emotion regulation	Process-outcome synthesis	ACT + routine support: (n = 36), mean change = 33.14, SD = 11.58	Routine support: (n = 36), mean change = 17.11, SD = 12.02	T2−T0 change score; T2 at week 12	Higher scores indicate greater use of positive emotion-regulation strategies; positive values favor ACT	Hedges g = 1.34 (95% CI 0.83 to 1.85)
Morthorst et al. [27], DSHI-Y NSSI episodes	Primary NSSI synthesis	ERITA + TAU: (n = 13), M = 3.46, SD = 6.62	TAU: (n = 14), M = 6.71, SD = 9.63	12-week post-treatment assessment; post-treatment scores; past-four-week NSSI episodes	Higher scores indicate more NSSI; negative values favor ERITA	Hedges g = −0.38 (95% CI −1.14 to 0.38)

Study and outcome	Role in synthesis	Intervention	Comparator	Time point and data used	Direction of scoring	Effect estimate
Falahati et al. [32], cognitive emotion regulation	Process-outcome synthesis	ACT: (n = 15), M = 55.43, SD = 6.21	No-training control: (n = 15), M = 48.73, SD = 6.25	Post-treatment following eight weekly sessions; post-treatment scores	Higher scores indicate better cognitive emotion regulation; positive values favor ACT	Hedges g = 1.05 (95% CI 0.28 to 1.82)
Bjureberg et al. [33], masked assessor-rated NSSI frequency	Reported separately; not pooled	IERITA + TAU: randomized (n = 84); post-treatment analysis (n = 77); median = 0, IQR = 0–4	TAU: randomized (n = 82); post-treatment analysis (n = 77); median = 3, IQR = 0–11	One-month post-treatment; model-based count outcome	Lower episode rate favors IERITA; IRR below 1 favors IERITA	Adjusted IRR = 0.34 (95% CI 0.20 to 0.57); not converted to Hedges g

Note. Values are those entered into the effect-size calculations, rather than the total numbers randomized, when outcome data were missing at the relevant assessment. Change scores were defined as T2 minus T0. For the Yuan et al. [15] NSSI outcomes, more negative change scores indicate larger reductions. The Bjureberg et al. [33] IRR was extracted directly from the adjusted count model and was not converted to a standardized mean difference. No effect size was reconstructed from significance levels alone. ACT = Acceptance and Commitment Therapy; ANSSIQ = Adolescent Non-Suicidal Self-Injury Questionnaire; CI = confidence interval; DSHI-Y = Deliberate Self-Harm Inventory–Youth version; ERITA = Emotion Regulation Individual Therapy for Adolescents; IERITA = internet-delivered Emotion Regulation Individual Therapy for Adolescents; IQR = interquartile range; IRR = incidence rate ratio; M = mean; NSSI = non-suicidal self-injury; SD = standard deviation; TAU = treatment as usual.
