# Supplementary material for: Acceptance-Based and ACT-Informed Interventions for Non-Suicidal Self-Injury in Adolescents: A Systematic Review and Exploratory Meta-Analysis
Source: Children (Basel). 2026 Jul 22;13(7):972. doi: 10.3390/children13070972 (PMC13406886; doi:10.3390/children13070972)
Supplement: Supplementary file 1 [file children-13-00972-s001.zip › Table S3.pdf]

Supplementary Table S3. Grading of Recommendations Assessment, Development and Evaluation (GRADE) certainty ratings for the principal outcome domains.

Outcome or evidence body	Evidence	Effect estimate	Risk of bias	Inconsistency	Indirectness	Imprecision	Publication bias	Certainty
Masked clinician-rated NSSI frequency: IERITA + TAU versus TAU	One RCT; 166 randomized, 154 included in the post-treatment analysis	IRR = 0.34 (95% CI 0.20 to 0.57)	Not serious. Some study-level concerns were present, but masked assessment of the primary outcome and prospective trial registration reduced the likelihood of material bias in the reported effect.	Not assessable; no downgrade. Only one study was available.	Serious. Evidence derives from one predominantly female Swedish sample and one specific multicomponent IERITA intervention.	Not serious for the reported short-term relative effect.	Undetected, although it cannot be excluded with a single study.	Moderate
Continuous NSSI frequency or severity	Two controlled studies; n = 99 analyzed	Hedges g = −0.45 (95% CI −0.85 to −0.05)	Serious: one small feasibility RCT and one retrospective non-randomized study	Not serious, but difficult to assess. The two point estimates were similar in direction and magnitude, although confidence in this	Serious: different interventions, comparators, measures, and assessment methods	Serious: only two studies and unstable estimates of between-study variance	Suspected because of the very small evidence base and unretrieved reports	Very low

Outcome or evidence body	Evidence	Effect estimate	Risk of bias	Inconsistency	Indirectness	Imprecision	Publication bias	Certainty
				judgment is limited by k = 2.				
Emotion-regulation/process outcomes	Two controlled non-randomized or semi-experimental studies; n = 102	Hedges g = 1.25 (95% CI 0.83 to 1.68)	Very serious	Not serious, but difficult to assess. Both estimates were in the same direction and of broadly similar magnitude, although only two studies were available.	Serious: process outcomes are indirect or surrogate outcomes rather than direct NSSI outcomes	Serious: only two small studies	Suspected	Very low
Feasibility and acceptability	Three ERITA studies; n = 72	Narrative synthesis; no pooled estimate	Very serious because two studies were uncontrolled and one was a small feasibility RCT	Serious because definitions and reporting differed across studies	Serious because samples and delivery formats were narrow	Serious because of small samples and descriptive estimates	Suspected	Very low
Adverse events and safety	Partial and non-uniform reporting across six primary studies; only	Not estimable; one RCT reported 5/84 versus	Very serious because safety outcomes were incompletely and potentially	Not assessable because outcomes and reporting methods differed substantially	Serious because key trials excluded adolescents with immediate,	Very serious because samples were small and	Not assessable; unfavorable or prematurely discontinued	Not rated because of insufficient evidence

Outcome or evidence body	Evidence	Effect estimate	Risk of bias	Inconsistency	Indirectness	Imprecision	Publication bias	Certainty
	one RCT reported suicide-attempt counts	9/82 suicide attempts	selectively reported		imminent, or severe suicide risk	serious events were rare	studies may be underrepresented	

Note. Certainty ratings were added post hoc during revision. An observed I^2 of 0% was not interpreted as evidence of homogeneity because estimates of heterogeneity are highly imprecise when k = 2. Nevertheless, no downgrade for inconsistency was applied to the two pooled quantitative outcomes because the study-level estimates were in the same direction and of broadly similar magnitude. Clinical and methodological differences between studies were addressed under indirectness. No upgrading for large effects was applied because the largest estimates arose from small or methodologically limited studies. Mediation findings from the IERITA trial were not assigned a separate certainty rating because they were based on a single secondary analysis and were not quantitatively commensurable with the pooled process outcomes. The reported suicide-attempt counts were not interpreted as a treatment effect because the trial was not designed or powered for this outcome. Abbreviations: IERITA = internet-delivered Emotion Regulation Individual Therapy for Adolescents; IRR = incidence rate ratio; NSSI = non-suicidal self-injury; RCT = randomized controlled trial; TAU = treatment as usual.
